# Trans- and Replantation of Teeth

**Published:** 1877-01

**Authors:** 


### TRANS- AND REPLANTATION OF TEETH.

Herr Rabatz has during the last three years made very interesting observations on this subject (v. *Memorabilien*, xxi. T. H. 3.; *Wein. Med. Presse*). In that period he has made eighty-four replantations; in forty-six of the cases the pulp was more or less diseased, while the periosteum was found normal. The teeth were drawn, stuffed, immediately replanted, and bandaged. Forty-one healed painlessly within three to four weeks. In the thirty-eight remaining cases periostitis existed. The condition of the pulp is of secondary importance. If diseased, it was scraped, but in all cases the tooth was stuffed before being replaced. The periosteum was scraped off in twenty of these teeth, and the points of the roots filled off slightly, while in the remaining eighteen the diseased membrane was left attached. In the former set seventeen were successful, of the latter only three. In one case both plans were tried at the same time, one tooth being replanted with the diseased membrane, while it was scraped from the other, both being shortened a little. The result was a perfect success. With regard to transplantation from one mouth to another, Rabatz thinks it will never become successful, except in extraordinary cases. The great difficulty lies in the form of the root. It is impossible to tell this before the tooth is drawn, and many might be pulled before one would be got to fit a particular alveolus.

